# Immune-Enhancing Formulas for Patients With Cancer Undergoing Esophagectomy: Systematic Review Protocol

**DOI:** 10.2196/resprot.7688

**Published:** 2017-11-17

**Authors:** Astrid Naranjo, Elizabeth Isenring, Laisa Teleni

**Affiliations:** ^1^ Faculty of Health Sciences and Medicine Bond University Gold Coast Australia

**Keywords:** immunonutrition, nutritional support, esophagectomy, postoperative infectious complications, immune-enhancing formula

## Abstract

**Background:**

Adult patients with an esophageal cancer can potentially be compromised with postoperative leaks or fistulae if patients’ nutritional status is in a vulnerable stage. Currently in Australia, there is a growing need for clinicians to know whether use of immune-enhancing formulas (IEFs) containing Arg, omega-3, and RNA are a cost-effective approach compared with isonitrogenous-isocaloric formulas to reduce postoperative infectious complications in esophagectomy patients. Since IEFs may carry higher costs, this has led to inconsistencies in practice among clinicians and hospitals.

**Objective:**

Our aim is to compile and present the most up-to-date nutrition evidence available regarding the provision of IEFs containing Arg, omega-3, and RNA to help clinicians develop an evidence-based nutrition care plan; identify available evidence of whether an esophagectomy patient should receive IEF; determine the cost-effectiveness and safety of such nutrition; and determine appropriate administration quantity and timing (pre-, peri-, or postesophagectomy).

**Methods:**

This review will include RCTs involving the use of IEFs enriched with Arg, omega-3 polyunsaturated fatty acids, and RNA in the pre-, peri-, or postoperative period (for at least 5-7 days) given orally or via enteral feeding tube, in adult cancer patients undergoing esophageal resection. Lower gastrointestinal, gastric, or head cancer surgery with parenteral nutrition or non-IEF or use of isolated immunonutrient (Arg vs omega-3 vs RNA) will be excluded. Primary outcome comprises postoperative infectious complications. Secondary outcomes (pre/postoperatively) consist of cost-effectiveness, length of stay, survival/mortality, quality of life, nutritional status, percentage of weight loss, and biochemical changes. The risk of bias will be independently assessed by the reviewers, using a domain-based evaluation tool. Blinding will be assessed for subjective and objective outcome measures. Publication bias will be visually assessed by funnel plots. A meta-analysis will be generated by the Review Manager 5.3 software and represented in forest plots.

**Results:**

The first results are expected in 2018. Outlining the protocol will ensure transparency for the completed review.

**Conclusions:**

This protocol for a systematic review and meta-analysis will enable a comprehensive appraisal of the literature to help determine whether overall institutional savings are associated with this approach. Findings will form a knowledge base relevant to stakeholders across the health system and researchers who are involved in decision making on evidence-based nutrition care plan pathways for patients undergoing esophagectomy, as well as the use of IEF, timing, and administration quantity.

**Trial Registration:**

PROSPERO Registration Number: CRD42017056908; http://www.crd.york.ac.uk/PROSPERO/ display_record.asp? ID=CRD42017056908 (Archived by WebCite at http://www.webcitation.org/6rLyeqaD6)

## Introduction

### Description of the Condition

Adult patients with an esophageal cancer or intractable strictures often require resection of the esophagus with complex anastomoses that can potentially be compromised with postoperative leaks or fistulae if patients’ nutritional status is in a vulnerable stage [1,2]. Additionally, pulmonary complications, especially pneumonia, are a major component of morbidity after an esophagectomy [3].

Although it is challenging to define and measure nutritional status in oncology, cancer-associated malnutrition seems to affect this population even in those overweight or well-nourished patients with a recent diagnosis [4]. Up to 85% of patients develop cancer-associated malnutrition, in which individual susceptibility depends on several factors such as the type, grade, and stage of cancer as well as the side effects of treatment modalities [5,6]. These factors may have a negative impact causing systemic anorexia combined with altered absorption of nutrients, unintentional weight loss, and depletion of lean body mass [4]. Moreover, pre-existing poor dietary habits, socioeconomic status, functional performance, nutritional impact symptoms, requirements for fasting, and inadequate nutritional therapy may affect nutritional intake as well, causing a progressive and widespread sarcopenia [6]. Because of this nutritional depletion, the risk of morbidity and mortality increases as well as the length of hospitalization and health care costs [7].

Currently in Australia, there is a growing need for clinicians to know whether the use of immune-enhancing formulas (IEFs) containing arginine (Arg), omega-3, and ribonucleic acid (RNA) is a cost-effective approach compared with isonitrogenous- isocaloric formula to reduce postoperative infectious complications (POIC) in esophagectomy patients [8]. Since these IEFs may carry higher costs, selective and cautious use with patients has led to inconsistencies among practitioners or hospitals, possibly because of insufficient targeted evidence-based guidelines advocating or supporting enteral immunonutrition (IN) in esophageal resection patients [9].

Thus, this systematic review will enable a comprehensive appraisal of the literature to assist in determining whether overall institutional savings are associated with this approach. This review also seeks to compile and present the most up-to-date available evidence on the provision of IEFs containing Arg, omega-3, and RNA, to help clinicians develop an evidence-based nutrition care plan. Furthermore, we aim to identify available evidence of whether an esophagectomy patient would be the ideal case to receive IN, and a cost-benefit analysis to determine appropriate administration quantity and timing (pre-, peri-, or postesophagectomy).

We aim to address the following research questions. In cancer patients undergoing esophageal resection and requiring postoperative nutritional support: (1) Is there sufficient quality evidence on perioperative IEF enriched with omega-3, Arg, and RNA to recommend it as routine practice among clinicians?, (2) Do IEFs enriched with omega-3, Arg, and RNA confer additional clinical benefits such as reducing the risk of POIC and improving patients’ health care outcomes compared to standard enteral nutrition?, and (3) Are IEFs enriched with omega-3, Arg, and RNA a cost-effective strategy to be considered by clinicians?

## Methods

### Types of Studies

This review will include randomized controlled trials (RCTs) on National Health and Medical Research Council level II intervention and use of IEFs in esophagectomy patients. The trials selected should contain IEFs (containing Arg, omega-3, and RNA) compared with standard enteral formulas (SEFs) either pre-, post-, or perioperative. Non-RCT intervention studies such as cohort studies will be excluded, since the potential risk of bias is higher. Likewise, we will exclude observational studies, abstracts, review papers, conference proceedings, and studies that are not English, Spanish, or Portuguese language.

Ethical approval is not required for the proposed systematic review and meta-analysis because the data used in this review will not involve the privacy of individual patients. Findings will be reported in line with the Preferred Reporting Items for Systematic Reviews and Meta-Analyses (PRISMA) [10]. As well, the manuscript will be submitted for publishing in a peer-reviewed journal or the findings presented at a relevant conference.

### Types of Participants

All patients 18 years or over undergoing surgical procedure for esophageal cancer will be included. Also, those included should be receiving IEF (containing a combination of Arg, omega-3, and RNA) pre-, peri-, or postoperatively for at least 5-7 days. Moreover, we will include all inpatient and outpatient/ambulatory patients in the health care facility where patients are having surgery (Textbox 1).

### Types of Interventions

The intervention will be the use of IEFs containing the immunonutrients Arg, omega-3 polyunsaturated fatty acid, and RNA provided either orally or via an enteral feeding tube. To be included, studies will have IEF given pre-, peri-, and/or postoperatively as an intervention. Co-intervention with other oral or parenteral substances will not be included. The control group should be receiving the traditional isonitrogenous-isocaloric SEF or polymeric nutritional supplements either orally or via enteral feeding tube.

### Primary and Secondary Outcomes

#### Primary Outcomes

The primary outcome will be POICs including wound infections or ﬁstulae formation, bacteremia, sepsis, anastomotic leakage, abscess and pulmonary complications, especially pneumonia or bronchopneumonia within the first 2 weeks of surgery and during the whole stay in hospital [3]. The measure will be as deﬁned by trial authors (categorical: present/absent or yes/no or continuous as number of cases or percentage of people with POIC).

**Participant inclusion and exclusion criteria.**

Inclusion criteria:

- Adult patients (≥18 years) undergoing an elective surgical procedure for esophageal cancer; perioperative patients
- Adult patients in various stages of the disease including I, II, III, and IV
- All patients (inpatient and outpatient) undergoing surgery
- Receiving perioperative IEF (Impact/other similar formulation: combination of Arg, omega-3, and RNA for at least 5-7 days)

Exclusion criteria:

- Lower GI cancer surgery, gastric, or head cancer surgery
- Patients with cancer having parenteral nutrition or enteral feeding with non-IEF or IEF with only one isolated nutrient (Arg vs omega-3 vs RNA)

#### Secondary Outcomes

We will consider the following secondary outcomes measured pre- and/or postoperatively:

- Health-related costs/cost-effectiveness/cost-benefit: defined by the authors
- Health care use: length of stay (LOS) measured in number of days; readmissions to acute care, subacute care or intensive care unit; re-operations measured in number of cases
- Survival/mortality: number/proportion of deaths related to treatment ˂18 months (long-term survival) [11]; number/proportion of deaths (≤30 days and/or in-hospital postoperatively) [11]
- Quality of life: health-related quality of life measures via a validated diagnostic tool such as the European Organisation for Research and Treatment of Cealth-related quality of life questionnaires (within the first 3, 6, and 24 weeks postoperatively [12]; including symptoms such as eating difficulties, reflux, dysphagia, and trouble with coughing [13]
- Nutritional status: classified as well-nourished or malnourished and measured by validated nutrition assessment tool within 2 weeks pre-operatively
- Percentage of weight loss: ˃5% weight loss in past month (1/12) and 1 week and/or 3 weeks postoperatively
- Biochemical changes: as per trial authors, including C-reactive protein levels (from ≤7 days prior surgery and first 2 weeks postoperatively)

### Searches

Searches for RCTs will be conducted systematically by the reviewers, with no publication year restriction. Non-English/Spanish/Portuguese studies might be excluded unless translations were provided or arranged. Also, original authors may be contacted for clariﬁcation and request of further data if trial reports seem unclear. Computerized searches will be performed for relevant published studies on the following databases from their inception until July 2017: the Cochrane Central Register of Controlled Trials (CENTRAL-The Cochrane Library), PubMed, EMBASE, CINAHL, LILACS, ClinicalTrials.gov, and the Trip (Turning Research into Practice) database. Subject strategies for databases in the search strategy will be modeled and designed for CENTRAL, CINAHL, and PubMed (Multimedia Appendix 1), which can be adjusted for other databases. Where appropriate, subject strategies will be combined with adaptations of the highly sensitive search strategy for identifying RCTs as described in the Cochrane Handbook for Systematic Reviews of Interventions Version 5.1.0 [14]. Additionally, during the searching process, consideration will be given to the different terminology used and the spelling of keywords as they may influence the identification of relevant studies.

The reference lists of identiﬁed publications will be scanned for further trials, and some trial authors may be contacted if required. Additional searches with Google Scholar, Google, and Bond University Library online will be conducted to retrieve remaining systematic reviews and meta-analyses pertinent to this review, in order to scan their reference lists for additional existing trials. A search of gray literature may be performed using the and .

### Data Collection and Analysis

#### Selection of Studies

The results of the searches will be combined as indicated in Multimedia Appendix 1. The titles and abstracts identified from the search will be screened and assessed independently by the reviewers against the inclusion criteria to remove those that are inappropriate. If the title or abstracts are inconclusive, further in-depth screening and evaluation against the inclusion criteria will be undertaken. The reviewers will record independently the reasons for study exclusion and will report them in a flow diagram according to the Preferred Reporting Items for Systematic Reviews and Meta-Analyses (PRISMA) format [10]. Disagreements over study selection will be resolved by discussion, and an external person may arbitrate if needed. Trial investigators shall be contacted if further information is required. All eligible references will be entered/collected into the bibliographic software package EndNote.

#### Data Extraction and Management

Data such as study design and setting, number of participants, outcome measure, and timing of IN initiation, route feeding, and total duration of IN will be autonomously extracted and organized on a matrix table (Multimedia Appendix 2). The meta-analysis data will also be pooled and organized on a matrix table (Multimedia Appendix 3). Reviewers will reach a consensus view by discussion. If further information is needed from an RCT, one of the reviewers may contact the nominated trial investigator/author. A plan of how data will be pooled and organized for the outcome measures analysis is shown in Multimedia Appendix 4.

#### Assessment of Risk of Bias

The risk of bias in the eligible studies will be independently assessed by the reviewers. Any disagreements will be resolved through discussion with an external arbitrator if required. Risk of bias assessment will be performed using the domain-based evaluation tool described by the  *Cochrane Handbook for Systematic Reviews of Interventions* [14].

Every RCT will be appraised according to the quality of 6 domains: (1) random sequence generation and allocation concealment (selection bias), (2) blinding of participants and personnel (performance bias), (3) blinding of outcome assessment (detection bias), (4) incomplete outcome data (attrition bias), (5) selective reporting (reporting bias), and (6) any other potential concerns to validity [14]. Blinding will be assessed separately for subjective (eg, nutritional status, LOS, quality of life) and objective (eg, percentage of weight loss, biochemical/immunological changes, mortality) outcome measures as the latter are less likely to be affected by knowledge of the treatment allocation group. Publication bias will be visually assessed by funnel plots as indicated by PRISMA [10].

Based on study reports, preliminary information will be collected in matrix tables to inform the risk of bias assessments (Multimedia Appendix 2). Trial authors shall be contacted to provide a study protocol or to clarify uncertainties where inconsistencies or unclear methodology were present during the risk of bias assessment.

A low risk of bias RCT will be considered if all or the majority of domains were gauged as adequate. RCTs will be considered as having a high risk of bias when one or more of the assessed domains within that trial were unclear or inadequate (unless original authors answer queries otherwise). The “risk of bias or internal validity” will be reported as part of the “characteristics and outcomes of identified studies” matrix (Multimedia Appendix 2) as well as a “risk of bias summary ﬁgure” presenting detailed judgments or explanations for all included studies in this review [2].

### Data Analysis and Synthesis

Categorical data will be presented as risk ratio and risk difference or odds ratio including their 95% confidence intervals (CI), which will be calculated/extracted for the analysis of most of the outcome measures apart from health care use (ie, LOS) and biochemical/immunological changes that are exclusively represented as continuous variables (Multimedia Appendix 4). Also, numbers needed to treat for beneﬁt or harm could be calculated as needed. Continuous data will be presented as mean, mean difference, or standardized mean difference with 95% CI, as applicable, excluding the nutritional status outcome measure, as it is represented exclusively as dichotomous variable. If a specific study does not report standard deviations, these will be calculated from the standard error and the sample size or the 95% CI. Finally, a *P* value of <.05 for both continuous and dichotomous variables will be considered statistically significant (Multimedia Appendix 4).

### Assessment of Heterogeneity

Heterogeneity must be considered from both a clinical and statistical perspective. On one hand, clinical expertise will be used to decide whether it would be meaningful to combine the studies based on the potential sources of heterogeneity for each outcome measure. The potential sources where the RCTs may not show the exact same result could depend on factors such as the patients’ demographics (eg, age: adult vs elderly), nutritional status baseline, type of surgery (invasive or less invasive), different doses and timing of pre- versus peri- versus postoperative IEF, definition of the outcome measures, as well as the timing for measuring those outcomes (Multimedia Appendix 5). Hence, clinical heterogeneity will be reported using subgroup (pre-, peri-, and postoperative subgroups using IEF; well-nourished vs malnourished patients’ subgroups). Sensitivity analysis might also be calculated by restricting the analysis to trials classified as having an overall low risk of bias, which will help determine whether excluding studies at high risk of bias affects the results of the meta-analysis [13,15,16].

On the other hand, statistical heterogeneity, which quantifies the variation due to heterogeneity and not due to chance across the RCTs, will be also checked through the statistical calculation of an I^2^ test [2]. Hence, values of 25%, 50%, or 75% will denote low, moderate, or high levels of heterogeneity respectively [2].

After that process, if the degree of clinical and statistical heterogeneity of studies are not excessive, a quantitative summary measures (meta-analysis) will be generated by the Review Manager (RevMan) 5.3 software and represented in forest plots [14]. Thus, the meta-analysis will characterize the effect of the dichotomous and continuous outcomes.

Where statistical pooling is not possible, the findings will be presented in narrative form or a table to describe the outcomes, different tools used to assess the outcomes listed for this review, or continuous data that can be pooled for meta-analysis.

### Confidence in Cumulative Estimate

The quality of evidence for all outcomes will be judged using the Grading of Recommendations Assessment, Development and Evaluation working group methodology. Therefore, the quality of evidence will be assessed across 5 domains: risk of bias, consistency, directness, precision, and publication bias. Additional domains may be considered where appropriate. High-quality evidence will be adjudicated when further research might be very unlikely to change the reviewer’s confidence in the estimate of effect; moderate quality if further research is likely to have a significant impact on the confidence and may change the estimate; low quality if further research is needed and is very likely to have an important impact and likely to change the estimate; and very low quality if there is considerable uncertainty about the estimate of effect.

## Results

The first results are expected in 2018. Outlining the protocol will ensure transparency for the completed review.

## Discussion

### Principal Considerations

Given the nature of esophageal cancer and the significant potential for nutritional deficiencies prior to resection as compared with other gastrointestinal (GI) pathologies, it seems appropriate that there should be a specific focus on the role of perioperative nutritional supplementation [17]. Research has shown that IEFs enriched with a combination of Arg, omega-3 fatty acids, including eicosapentaenoic acid (EPA) and docosahexaenoic acid (DHA), RNA, and antioxidants [18], can act pharmacologically on the immune system and potentially improve the patient’s immune response [9]. Furthermore, providing patients with peri-operative IEF has shown to favorably modulate the inﬂammatory, metabolic, and immune response after upper GI surgery [19,20]. These effects generated on the immune system modulated through specific nutrients are called immunonutrition.

IEFs enriched with a combination of immunonutrients (Arg, omega-3, and RNA) are well known to Australian clinicians. However, IN is not consistently used with cancer patients undergoing esophageal resection as standard practice [21]. Since this IEF offers the combination of immunonutrients in one product, it has been implemented in a wide range of surgical and trauma patients [22] to significantly reduce POICs by 39%-61%, LOS by 2 days [23], antibiotic use, and health-related costs [18]. Several studies have shown positive outcomes among GI surgical patients, where anastomotic leaks were 46% less prevalent when these IEFs were part of the pre-operative nutrition care plan [3,18,21,23,24]. Additionally, it has been found that LOS and POIC in malnourished (upper and lower) GI cancer patients significantly decreased when a “5-7-day peri-operative approach” was implemented with IEF over SEF [18,25,26]. Thus, nutrition support of surgical patients with malnutrition should not only include the adequate quantity but also the nutrient quality, type, and prescription timeframe to support the patient’s recovery, postoperative outcomes, and reduce health care costs [20,27-29]. In terms of health care cost-benefit analysis, the implementation of IN in surgical patients resulted in savings of US $3300 per patient based on the reduction of POIC rates [22] and reported savings of up to US $6000 per patient, based on the shortening of LOS [16], showing that enteral IEFs are a beneficial and cost-effective intervention.

### Conclusion

To date, there have been several systematic reviews and meta-analyses of RCTs undertaken to determine the effectiveness of IN in elective upper and lower GI surgical practice [7,16,21,30]. However, the effect of a particular combination of immunonutrients (Arg, omega-3, and RNA) and its overall effect on postesophagectomy outcomes have not been systematically reviewed. Hence, there is a lack of sufficient, recent clinical evidence supporting the use of IEFs in esophagectomy patients. Evaluation of the studies identiﬁed in this review has merit because the review will aim to provide the most up-to-date evidence-based nutrition statements regarding pre-, peri-, and postoperative enteral IN effects on postesophagectomy outcomes.

## Appendix

Multimedia Appendix 1 Search terms and results of the search strategy.

Multimedia Appendix 2 Characteristics of studies including immunonutrition for esophagectomy.

Multimedia Appendix 3 Characteristics of RCTs included in meta-analysis of immune-enhancing formula versus standard enteral formula.

Multimedia Appendix 4 Outcome measures analysis table.

Multimedia Appendix 5 Table of the planned statistical analysis for the meta-analysis.

## Abbreviations

Arg: arginine
CI: confidence intervals
GI: gastrointestinal
IEF: immune-enhancing formula
IN: immunonutrition
LOS: length of stay
POIC: postoperative infectious complication
RCT: randomized controlled trial
RNA: ribonucleic acid
SEF: standard enteral formula
