# Metadata Correction: Immune-Enhancing Formulas for Patients With Cancer Undergoing Esophagectomy: Systematic Review Protocol

**DOI:** 10.2196/resprot.9615

**Published:** 2018-04-27

**Authors:** Astrid Naranjo, Elizabeth Isenring, Laisa Teleni

**Affiliations:** ^1^ Faculty of Health Sciences and Medicine Bond University Gold Coast Australia

In the paper by Astrid Naranjo et al, “Immune-Enhancing Formulas for Patients With Cancer Undergoing Esophagectomy: Systematic Review Protocol” (JMIR Res Protoc 2017;6(11):e214), mistakes were made when listing the authors’ degrees. Laisa Teleni’s highest qualification was incorrectly listed as PhD. The correct degrees for Laisa Teleni are “BBiomedSci (Hon), MND”. Elisabeth Isenring’s degrees were incorrectly listed as “BHSc (Nut & Diet, Hons 1, GradCertHighEd)”. The correct degrees for Elisabeth Isenring are “BHSc (Nut & Diet, Hons 1), PhD”. The affiliations of the authors have not been changed.

The corrected article will appear in the online version of the paper on the JMIR website on April 27, 2018, together with the publication of this correction notice. Because this was made after submission to PubMed, Pubmed Central, and other full-text repositories, the corrected article also has been re-submitted to those repositories.

