# Supplementary material for: Immune-Enhancing Formulas for Patients With Cancer Undergoing Esophagectomy: Systematic Review Protocol
Source: JMIR Res Protoc. 2017 Nov 17;6(11):e214. doi: 10.2196/resprot.7688 (PMC5712009; doi:10.2196/resprot.7688)
Supplement: Multimedia Appendix 1 [file resprot_v6i11e214_app1.pdf]

Appendix 1 Search terms and results of the search strategy

Database	Search terms	Hits	Date
PubMed	(oesophagectomy OR esophagectomy OR oesophagus resection) AND (nutrition support OR enteral nutrition OR nutrition supplementation OR supplements OR immunonutrition or immune-enhancing formula	34	2/2/17
	(("oesophagectomy"[All Fields] OR "esophagectomy"[MeSH Terms] OR "esophagectomy"[All Fields]) OR ("oesophagectomy"[All Fields] OR "esophagectomy"[MeSH Terms] OR "esophagectomy"[All Fields]) OR (("oesophagus"[All Fields] OR "esophagus"[MeSH Terms] OR "esophagus"[All Fields]) AND resection[All Fields])) AND (("nutritional support"[MeSH Terms] OR ("nutritional"[All Fields] AND "support"[All Fields]) OR "nutritional support"[All Fields] OR ("nutrition"[All Fields] AND "support"[All Fields]) OR "nutrition support"[All Fields]) OR (("nutritional status"[MeSH Terms] OR ("nutritional"[All Fields] AND "status"[All Fields]) OR "nutritional status"[All Fields] OR "nutrition"[All Fields] OR "nutritional sciences"[MeSH Terms] OR ("nutritional"[All Fields] AND "sciences"[All Fields]) OR "nutritional sciences"[All Fields]) AND supplementation[All Fields]) OR supplements[All Fields] OR immunonutrition[All Fields] OR (immune-enhancing[All Fields] AND ("food, formulated"[MeSH Terms] OR ("food"[All Fields] AND "formulated"[All Fields]) OR "formulated food"[All Fields] OR "formula"[All Fields])) OR ("food, formulated"[MeSH Terms] OR ("food"[All Fields] AND "formulated"[All Fields]) OR "formulated food"[All Fields] OR "formula"[All Fields]))	321 (filter: humans) 81 (filter: clinical trials and Reviews)	2/2/17
	(esophageal cancer OR oesophageal cancer OR neck cancer OR neck neoplasm* OR oesophagectomy OR esophagectomy OR oesophagus resection) AND (IEF OR nutrition support OR nutrition supplementation OR supplements OR immunonutrition OR immune-enhancing formula OR formula)	668 (filter: humans, Clinical trials and rv)	2/2/17
	(("oesophageal cancer"[All Fields] OR "esophageal neoplasms"[MeSH Terms] OR ("esophageal"[All Fields] AND "neoplasms"[All Fields]) OR "esophageal neoplasms"[All Fields] OR ("esophageal"[All Fields] AND "cancer"[All Fields]) OR "esophageal cancer"[All Fields]) OR ("oesophageal cancer"[All Fields] OR "esophageal neoplasms"[MeSH Terms] OR ("esophageal"[All Fields] AND "neoplasms"[All Fields]) OR "esophageal neoplasms"[All Fields] OR ("esophageal"[All Fields] AND "cancer"[All Fields]) OR "esophageal cancer"[All Fields]) OR ("head and neck neoplasms"[MeSH Terms] OR ("head"[All Fields] AND "neck"[All Fields] AND "neoplasms"[All Fields]) OR "head and neck neoplasms"[All Fields] OR ("neck"[All Fields] AND "cancer"[All Fields]) OR "neck cancer"[All Fields]) OR (neck neoplasm[All Fields] OR neck neoplasma[All Fields] OR neck neoplasms[All Fields]) OR ("oesophagectomy"[All Fields] OR "esophagectomy"[MeSH Terms] OR "esophagectomy"[All Fields]) OR ("oesophagectomy"[All Fields] OR "esophagectomy"[MeSH Terms] OR "esophagectomy"[All Fields]) OR (("oesophagus"[All Fields] OR "esophagus"[MeSH Terms] OR "esophagus"[All Fields]) AND resection[All Fields])) AND (IEF[All Fields] OR ("nutritional support"[MeSH Terms] OR ("nutritional"[All Fields] AND "support"[All Fields]) OR "nutritional support"[All Fields] OR ("nutrition"[All Fields] AND "support"[All Fields]) OR "nutrition support"[All Fields]) OR (("nutritional	670 (filter human, clinical trial, and RVs)	2/2/17

	status"[MeSH Terms] OR ("nutritional"[All Fields] AND "status"[All Fields]) OR "nutritional status"[All Fields] OR "nutrition"[All Fields] OR "nutritional sciences"[MeSH Terms] OR ("nutritional"[All Fields] AND "sciences"[All Fields]) OR "nutritional sciences"[All Fields]) AND supplementation[All Fields]) OR supplements[All Fields] OR immunonutrition[All Fields] OR (immune-enhancing[All Fields] AND ("food, formulated"[MeSH Terms] OR ("food"[All Fields] AND "formulated"[All Fields]) OR "formulated food"[All Fields] OR "formula"[All Fields])) OR ("food, formulated"[MeSH Terms] OR ("food"[All Fields] AND "formulated"[All Fields]) OR "formulated food"[All Fields] OR "formula"[All Fields])) AND ((Clinical Trial[ptyp] OR Review[ptyp]) AND "humans"[MeSH Terms])		
PUbmed	(esophagus cancer OR esophagus resection OR oesophagectomy OR (oesophagus AND cancer) OR oesophagus OR (oesophagus AND resection)) AND (perioperative period OR postoperative OR preoperative period OR perioperative) AND ((nutrition AND care) OR immunonutrition OR (immunol AND enhancing AND formula) OR enteric feeding) AND (postoperative complication* OR length of stay OR los OR pneumonia OR leakage)	44 (filter Clinical trial, human, Rvs)	2/2/17
CINAHL	(MH "Esophageal Neoplasms" OR "esophagus resection" OR oesophagectomy OR esophagectomy) AND ((MH "Perioperative Care") OR (MH "Postoperative Care") OR (MH "Preoperative Care") OR (MH "Intraoperative Care")) AND ("immunonutrition" OR ((MH "Arginine") AND ((MH "Fatty Acids, Omega-3") OR (MH "Eicosapentaenoic Acid") OR (MH "Docosahexaenoic Acids")) AND (MH "RNA"))	5	2/2/17
	(MH "Esophageal Neoplasms" OR "esophagus resection" OR oesophagectomy OR esophagectomy) AND ((MH "Perioperative Care") OR (MH "Postoperative Care") OR (MH "Preoperative Care") OR (MH "Intraoperative Care")) AND ("immunonutrition" OR nutrition support)	2	2/2/17
	(MH "Esophageal Neoplasms" OR "esophagus resection" OR oesophagectomy OR esophagectomy) AND ("immunonutrition" OR nutrition support)	14	2/2/17
CENTRAL	(esophageal cancer OR oesophageal cancer OR neck cancer OR neck neoplasm* OR oesophagectomy OR esophagectomy OR oesophagus resection) AND (IEF OR nutrition support OR nutrition supplementation OR supplements OR immunonutrition OR immune-enhancing formula OR formula)	9	2/2/17
CENTRAL	(oesophagectomy OR esophagectomy OR oesophagus resection OR esophagus cancer OR oesophagus cancer) AND (perioperative immunonutrition supplementation OR immunonutrition OR immune-enhancing formula)	6	2/2/17
Embase	('esophagus cancer'/exp OR 'esophagus resection'/exp OR oesophagectomy OR oesophagus cancer OR oesophagus resection) AND ('perioperative period'/exp OR 'postoperative care'/exp OR 'preoperative period'/exp) AND (immunonutrition OR immuno enhancing formula OR 'enteric feeding'/exp)	108	2/2/17
	'esophagus cancer'/exp OR 'esophagus resection'/exp OR 'oesophagectomy'/exp OR oesophagectomy OR 'oesophagus'/exp OR oesophagus AND ('cancer'/exp OR cancer) OR 'oesophagus'/exp OR oesophagus AND ('resection'/exp OR resection) AND ('perioperative period'/exp OR 'postoperative care'/exp OR 'preoperative period'/exp) AND ('immunonutrition'/exp OR immunonutrition OR 'immuno'/exp OR immuno AND enhancing AND formula OR 'enteric	50	2/2/17

	feeding'/exp) AND ('postoperative complication' OR los OR 'pneumonia' OR 'leakage')		
TRIP database	(oesophagectomy OR esophagectomy OR oesophagus resection OR esophagus cancer OR oesophagus cancer) AND (perioperative immunonutrition supplementation OR immunonutrition OR immune-enhancing formula)	RCT & clinical trials & SR 21	2/2/17
