# Supplementary material for: Immune-Enhancing Formulas for Patients With Cancer Undergoing Esophagectomy: Systematic Review Protocol
Source: JMIR Res Protoc. 2017 Nov 17;6(11):e214. doi: 10.2196/resprot.7688 (PMC5712009; doi:10.2196/resprot.7688)
Supplement: Multimedia Appendix 2 [file resprot_v6i11e214_app2.pdf]

Appendix 2 Characteristics of studies included IN for oesophagectomy

Reference number	Citation Study	Design & setting	Participants	Intervention aim & description OR Indicator/Issue of interest	Attrition	Type of IEF	NHMRC level of evidence	Internal validity (Study quality*)	Outcomes
1	**Example** [40]	Diagnostic, Observational, cross-sectional and case-control study[1] (for test accuracy) in oncology ward of a private tertiary Australian hospital	71 cancer patients aged 18-92 y	Scored PG-SGA questionnaire, comparison of scored PG-SGA with subjective global assessment (SGA), sensitivity, specificity on cancer patients aged 18 – 92 y	N/A	Impact Arg RNA ω-3PUFA	Level III-2	**Strong recommendation**<br>**Low quality evidence**<br>**Moderate risk of bias**<br><br>- High risk of Spectrum Bias<br>-Potential methodology (not standardized) bias<br>- Potential risk of observer/detection bias<br>- Potential risk of Diagnostic Review Bias	Concurrent validity of PG-SGA as assessment tool in oncology patients
			**Overall Grading Body of evidence**		**Recommendation**				
