# Supplementary material for: Immune-Enhancing Formulas for Patients With Cancer Undergoing Esophagectomy: Systematic Review Protocol
Source: JMIR Res Protoc. 2017 Nov 17;6(11):e214. doi: 10.2196/resprot.7688 (PMC5712009; doi:10.2196/resprot.7688)
Supplement: Multimedia Appendix 3 [file resprot_v6i11e214_app3.pdf]

# Appendix 3 Characteristics of RCTs included in meta-analysis of IEF versus standard EN

| Author (Year) [Ref] | Design & setting | NHMRC level of evidence | Participants | Number of participants Intervention | Standard control group | Outcome measured and Timing of IMN initiation | Mode of enteral feeding | Total duration of nutritional support (days) | Internal validity (Study quality*) |
|---|---|---|---|---|---|---|---|---|---|
| **1 Example** [40] | RCT in oncology ward of a private tertiary Australian hospital | Level III-2 | 90 | 46 | 44 | Impact Arg RNA ω-3PUFA<br><br>Post op | NJ | **4** | **Strong recommendation Low quality evidence Moderate risk of bias** |
| | | | | | | | | | |
| | | | | | | | | | |
| | | | | | | | | | |
| **Overall Grading Body of evidence** | | | | | | | | | |
