# Supplementary material for: Immune-Enhancing Formulas for Patients With Cancer Undergoing Esophagectomy: Systematic Review Protocol
Source: JMIR Res Protoc. 2017 Nov 17;6(11):e214. doi: 10.2196/resprot.7688 (PMC5712009; doi:10.2196/resprot.7688)
Supplement: Multimedia Appendix 4 [file resprot_v6i11e214_app4.pdf]

Appendix 4 Outcome Measures Analysis Table

Outcome	Type of variable	Potential outcomes measures	Data pooling	Data extraction	Timeframe points in time
[Name & Description]	*Continuous or dichotomous*	*What unit/s is the outcome measured in?*	*How will you pull data?*	*What data needs to be extracted for analysis?*	
**Primary outcome**					
**Postoperative infectious complications (POIC)**	Continuous<br><br>or<br><br>Dichotomous	Continuous: Incidence (%) or Number of cases with POIC or incidence of infections (% of reduction)<br><br>Dichotomous: yes or no POIC	All infections pulled together (POIC)	Continuous<br>The proportion of patients experiencing POIC mean differences, mean/median and SD with 95%CI + p value<br><br>Dichotomous<br>RR or OR and 95% CIs/p value from total number of participants that got POIC	Within the first 2 weeks of surgery and/or during the whole stay in hospital
**Secondary outcomes**					
**Health-related costs/cost-effectiveness**	Continuous<br><br>Or<br><br>Dichotomous	:Continuous<br>Cost of the intervention IEF in monetary units (i.e: $AU) and/or percentage (%) or net savings per patient compared with SEF<br><br>**Or**<br>Diagnosis-Related Group (DRG) reimbursement rate<br><br>Dichotomous<br>Expected health gain measured in natural units such as number	Group by similar outcome	Continuous<br>Percentage (%) of savings or mean cost per randomized patient + mean difference (MD) +<br>P value (if available)<br><br>Economic analysis and cost-effectiveness summarized in a narrative form (relationship with Postop morbidity and LOS)	N/A (associated with Postoperative morbidity and LOS outcomes)

Outcome	Type of variable	Potential outcomes measures	Data pooling	Data extraction	Timeframe points in time
		of lives saved<br>Or<br>Cost-benefit relationship with IEF: yes or no?<br><br>Or defined by authors<br><br>NB: QALYs would be ideal to find in studies but it might not be calculated for many studies			
**Healthcare use**<br><br>✓ Length of stay (LOS)<br>✓ Readmissions to acute care, sub-acute care or ICU<br>✓ Reoperations	Continuous	**LOS** measured in number of days<br><br>**Readmissions and reoperations** measured in number of cases	Number of days/cases All LOS days together, all readmissions and reoperation cases together	Number of cases/days represented by their mean + SD/SEM/95%CI and p value	Within 18 months post-surgery
**Survival or Mortality**	Continuous<br><br>Or<br><br>Dichotomous	**Mortality within** 18 months<br>And<br><br>**Postoperative mortality** Measured by number of cases Or YES/NO death	Group by similar cause/type of complication leading to death	Continuous: Number or proportion of cases represented by the SD/SEM/95%CI + p value<br><br>Dichotomous: RR, Hazard ratios with 95% CI and p value<br>for death adjusted for age; sex; comorbidity; and cancer stage, location, and histology	Mortality within 30-day and/or in-hospital post operatively<br><br>And<br>Within 18 months post-surgery
**Quality of life (QoL)**<br><br>As assessed by any validated tool	Any Validated tool continuous or categorical outcome or both.	Measured via a validated diagnostic tool/survey such as Health-	QoL domains: talking, pain, swallowing, eating	Mean difference and mean change Reporting on p value or 95% CIs for change	Within the first 3, 6 and 24 weeks postoperatively

Outcome	Type of variable	Potential outcomes measures	Data pooling	Data extraction	Timeframe points in time
	Dichotomous if each domain outcome is reported as Yes or no for talking, pain, swallowing<br><br>NB: If collected (report derived scores if available)	Related Quality of Life Measures	difficulties, reflux, dysphagia, and trouble with coughing.	Categories prevalence, p value<br><br>Odds ratios (ORs) with 95% CIs	
**Nutritional status**	Dichotomous	Dichotomous: Malnourished (moderate and severe) Well-nourished Defined and measured by Via PG-SGA, MNA, MUST, SGA	Subgroups Well-nourished, malnourished	Dichotomous RR/ OR of nutritional status from total number of participants	Within 2 weeks preoperatively to see the risk of complications associated with malnutrition
**Weight loss**	Dichotomous (yes or no)<br><br>or<br><br>Continuous (amount of FFM lost)	Weight loss > 5% of weight loss in past month (1/12) Or defined by authors (i.e. continuous amount of fat free mass lost)	Subgroup Patients with % > 5% wt loss Patients with <5% wt loss	Dichotomous RR or OR from total number of participants loss wt >5% + p value<br><br>Continuous mean of FFM loss (kg) + p value	Weight loss > 5% of weight loss in past month (1/12) and 1 week and/or 3 weeks after surgery
**Biochemical/ immunological changes**	Continuous	C-reactive protein (CRP), Immunoglobulins	Group by similar outcome units of each test	Mean, SD, mean difference (MD), p value	Pre-surgery and within first 2 weeks post-surgery

NB: some of the data type & measures were observed/retrieved from (M. Braga & Gianotti, 2005; Marco Braga et al., 2005; Drover et al., 2011; Mudge, Isenring, & Jamieson, 2011; Sakurai et al., 2007; Senkal et al., 1999; Waitzberg et al., 2006; Wong & Aly, 2016; Djärv et al, 2009; Rouvelas et al, 2005)
