# Supplementary material for: Immune-Enhancing Formulas for Patients With Cancer Undergoing Esophagectomy: Systematic Review Protocol
Source: JMIR Res Protoc. 2017 Nov 17;6(11):e214. doi: 10.2196/resprot.7688 (PMC5712009; doi:10.2196/resprot.7688)
Supplement: Multimedia Appendix 5 [file resprot_v6i11e214_app5.pdf]


Appendix 5 Table of the planned Statistical analysis for the Meta-Analysis

Dependent Variable	Comparator/ independent variable	Effect measure	Potential sources of heterogeneity
Outcome measure	*Intervention/ exposure*	*How will the overall effect be analyzed? Ie risk ratio, mean difference or standardized mean difference*	*Why might the studies not show the exact same result?*
**Primary outcome**			
**Postoperative infectious complications (POIC)**	Enteral immune-enhancing formula (Arg, w-3, RNA) Vs usual care Standard enteral formula (isonitrogenous-isocaloric)	For continuous The proportion of patients experiencing POIC mean differences mean/median and SD, 95%CI<br><br>Dichotomous RR, 95% CI, fixed effect size from total number of participants that got POIC	✓ Risk of bias, differences (blinding, performance, reporting)<br>✓ Low methodological quality of trials present<br>✓ Age (age/ adult versus elderly)<br>✓ Type of surgery (invasive or less invasive)<br>✓ Baseline nutritional status<br>✓ Dosage of IEF<br>✓ Timing of IEF provided
**Secondary outcomes**			
**Health-related costs/cost-effectiveness**	Enteral IEF (Arg, w-3, RNA) Vs isonitrogenous-isocaloric usual care SEF	% of savings or mean difference (MD) + P value if available, economic analysis and cost-effectiveness summarized in a narrative form (relationship with Postop morbidity and LOS)	✓ Nutritional status at baseline<br>✓ Age (age/adult versus elderly)<br>✓ Type of surgery (invasive or less invasive)<br>✓ Baseline nutritional status<br>✓ Dosage & timing of IEF
**Healthcare Use**<br>✓ Length of stay (LOS)<br>✓ Readmissions to acute care, sub-acute care or ICU<br>✓ Reoperations	As above	Number of cases/days represented by their mean+ mean difference + SD/95% CI effect size (ES) (random or fixed)	✓ Low methodological quality of trials present<br>✓ Age (age/ adult versus elderly)<br>✓ Type of surgery (invasive or less invasive)<br>✓ Baseline nutritional status<br>✓ Dosage of IEF<br>✓ Timing of IEF provided
**Survival/mortality**	As above	Dichotomous: RR , hazard ratios 95% CI, overall size effect fixed<br><br>Or<br><br>Number of cases/days represented by their mean + SD/SEM/95%CI and p value narrated form	✓ Age (age/ adult versus elderly)<br>✓ Type of surgery (invasive or less invasive)<br>✓ Baseline nutritional status<br>✓ Dosage of IEF<br>✓ Timing of IEF provided
**Quality of life (QoL)**	As above	Continuous: Number of cases represented by the	✓ Risk of bias, differences

Dependent Variable	Comparator/ independent variable	Effect measure	Potential sources of heterogeneity
**As assessed by any validated tool**		SD/SEM/95%CI + p value<br><br>Dichotomous:<br>RR , 95% CI and p value	(blinding, performance, reporting)<br>✓ Low methodological quality of trials present<br>✓ Age (age/ adult versus elderly)<br>✓ Type of surgery (invasive or less invasive)<br>✓ Baseline nutritional status
**Nutritional status**	As above	Mean and mean difference<br><br>Categories prevalence, p value<br><br>Reporting on p value or confidence intervals for change	✓ Risk of bias, differences (blinding, performance)<br>✓ Low methodological quality of trials present<br>✓ Age (age/ adult versus elderly)<br>✓ Pre-operative diet and Socioeconomic status of patients<br>✓ Assessment tool used – interpretation subjective<br>✓ Stage of disease of patients
**Weight loss**	As above	Dichotomous RR or OR from total number of participant's loss wt >5% + p value<br>Effect size<br><br>Continuous: Mean of FFM loss (kg) + p value	As above
**Biochemical changes**	As above	Mean, SD, mean difference (MD), p value<br>May be reported in a narrative form if few studies report it	✓ Risk of bias, differences (blinding, performance)<br>✓ Type of surgery (invasive or less invasive)<br>✓ Diagnostic tool used-method<br>✓ Stage of disease/stress of patients

RR: risk ratio; OR: odds ratio; SD: standard deviation; FFM: fat free mass; CI: confidence interval
